# Bacterial Contamination of Environmental Surfaces of Veterinary Rehabilitation Clinics

**DOI:** 10.3390/ani14131896

**Published:** 2024-06-27

**Authors:** Henry G. Spratt, Nicholas Millis, David Levine, Jenna Brackett, Darryl Millis

**Affiliations:** 1 Department of Biology, Geology, and Environmental Science, University of Tennessee at Chattanooga, Chattanooga, TN 37403, USA; jennabrac@gmail.com; 2 Department of Small Animal Clinical Sciences, University of Tennessee College of Veterinary Medicine, Knoxville, TN 37996, USA; nickmillis1996@gmail.com (N.M.); boneplate@aol.com (D.M.); 3 Department of Physical Therapy, University of Tennessee at Chattanooga, Chattanooga, TN 37403, USA; david-levine@utc.edu

**Keywords:** veterinary rehabilitation, canine rehabilitation, bacterial contamination, clinic environmental surfaces, staphylococci, MRSA, *Clostridium difficile*

## Abstract

**Simple Summary:**

This study was conducted to provide background data on the potential bacterial contamination of environmental surfaces in veterinary rehabilitation clinics. Knowledge of bacterial contamination in these clinics is important for effective rehabilitative outcomes for veterinary patients. This is particularly true when surgery has occurred prior to the rehabilitation. There is abundant evidence from human surgical recovery and rehabilitation that surgical site infections (SSIs) represent a major type of healthcare-associated infection. With human patients, *Staphylococcus* spp. (including the methicillin *S. aureus* strain—MRSA) are often associated with SSIs. For human patients, SSIs have been correlated with environmental bacterial contamination in clinics. Whether SSIs in veterinary patients are more prevalent when clinic environments are contaminated with potentially pathogenic bacteria has not been the subject of any substantial research to date. The purpose of this study is to provide background data on presumptive environmental surface bacterial contamination by potential pathogens in veterinary rehabilitation clinics. Our data suggest that bacterial contamination in these clinics is widespread. We have detected potential pathogens, including MRSA, *S. pseudintermedius*, various enteric bacteria, and *Clostridium difficile*, in the clinics sampled. These bacterial species may pose a problem to either clinic veterinary patients or human caregivers.

**Abstract:**

The presence of potentially pathogenic bacteria on veterinary clinic surfaces may be problematic. In this study, we collected swab samples (Fisherbrand, double transport swabs with Stuart’s liquid medium) and water samples from five veterinary rehabilitation clinics. Swabs and water samples were transported to a microbiology lab for processing. At the lab, swabs were used to inoculate Hardy’s Cdiff Banana Broth (for *Clostridium difficile* [Cdiff]) and five different types of bacterial growth media, including Hardy CHROM MRSA agar (methicillin-resistant *Staphylococcus aureus* [MRSA] and *S. pseudintermedius* [SIM]), mannitol salt agar (*S. aureus* [SA]), eosin methylene blue agar (enterics [ENT]), *Pseudomonas* isolation agar (*Pseudomonas* spp. [PS]), and tryptic soy agar [TSA] (non-specific). The most prominent presumptive species cultured was Cdiff (on nearly 55% of swabs). *Bacillus* spp. and enteric bacteria were encountered on nearly 35% of swabs, with MRSA and SIM on just over 10% of swabs. The most contaminated sample site was harnesses/life jackets used with the underwater treadmill (33% of swabs). The underwater treadmill water had total bacterial counts from 1,600 to 2,800 cfu/mL. Of all presumptive bacterial species detected, SIM tends to be more pathogenic for dogs. Targeted cleaning/disinfecting in these clinics could help reduce risks for both animals and caregivers utilizing these clinics.

## 1. Introduction

Healthcare-associated infections (HAIs) are a concern for patients treated in medical facilities, including veterinary physical rehabilitation facilities [1,2,3]. Pathogens can be especially concerning for postoperative patients or those who are hospitalized for prolonged time periods. These concerns are important in facilities for human and animal patients, with potential transmission from equipment or other external surfaces to the patients as a possible cause of HAIs [4,5]. In human medicine, certain pathogens tend to be of the most concern for serious medical problems, including *Clostridium difficile* [Cdiff], *Staphylococcus aureus* ([SA], especially methicillin-resistant variants [e.g., MRSA]), *Staphylococcus pseudintermedius* [SIM], and multidrug-resistant, Gram-negative rods (e.g., enteric bacteria [ENT] that may include *Escherichia* spp., *Enterobacter* sp. and *Klebsiella* sp.). While not necessarily of equal concern in human and veterinary clinics, the presence of these bacterial species on surfaces in any clinic represents contamination that could lead to HAIs [6].

Veterinary rehabilitation clinics represent a specialty of veterinary medicine in which animals that are recovering from an injury or surgery receive rehabilitation similar to human physical therapy. These rehabilitation clinics include therapeutic modalities, such as therapeutic ultrasound and lasers [7], and exercise equipment, such as treadmills, underwater treadmills, therapy balls, and other unstable surfaces for exercise [8]. These clinics commonly see postoperative patients with healing incisions and animals that are paralyzed or have limited mobility. Avoiding the contamination of surgical site wounds and preventing infections from shared equipment is important to consider in veterinary rehabilitation.

The bacterial contamination of surfaces in veterinary rehabilitation clinics can be problematic for both the animals being treated and the human caretakers. For example, Cdiff is a pathogen that can cause severe disease in humans. Infection by Cdiff can cause severe diarrhea and pseudomembranous colitis [9,10]. Although Cdiff has been isolated from dogs, a link between human infection with Cdiff and companion animals is not well documented and should be further explored [11,12,13]. 

Staphylococci, including SA and SIM, are opportunistic pathogens in both humans and animals [14,15,16,17]. Typically, SA infections have been successfully treated with topical or systemic courses of antibiotics [15]. However, antibiotic-resistant staphylococcal strains, including MRSA, represent an increasing problem throughout all of healthcare. These pathogens may also be isolated from companion animal veterinary patients, in addition to other pathogens that affect animals, and can also be transmitted from humans to animals.

Pathogens with known transmission between humans and animals include SA, MRSA, and SIM [16,17]. Orden et al. [11] isolated Cdiff from 12% of the dogs they studied. In a similar study, Álvarez-Perez et al. [6] found Cdiff infections in 5% of the dogs they sampled. Cdiff can cause enteritis, diarrhea, and hemorrhagic diarrhea in both humans and dogs [11,18]. Since Cdiff can be isolated from dogs, a major concern is the possible transfer of this pathogen to human caregivers. Since Cdiff spores may be found on the environmental surfaces of both human and animal clinics, knowledge of this contamination in veterinary clinics could be important to prevent HAIs in both humans and animals.

Staphylococcal infections in canine patients are often the result of surgical procedures and may cause moderate to severe morbidity [19]. With the continued impact of MRSA in human medicine and the close contact between humans and household pets, there has been an increase in MRSA in household pets [20]. Most infections associated with MRSA in veterinary patients are community-acquired and often transmitted from pet owners to their pets [21]. The prevalence of MRSA infections in veterinary patients ranges from 0 to 9% and may result in different symptoms related to skin and soft tissue infections, especially surgical site wounds, otitis, and pyoderma [15,21,22]. 

*Staphylococcus pseudintermedius* is a more common form of *Staphylococcus* spp. isolated from dogs and is responsible for infections such as pyoderma and otitis [23,24]. Starting in 2005, *S. pseudintermedius* became a novel species within the *S. intermedius* group of staphylococci [25]. It was formerly believed that dogs were colonized by *S. intermedius,* but in fact, the most common staphylococcal opportunistic pathogen associated with dogs is *S. pseudintermedius* [26,27,28]. Similar to other *Staphylococcus* spp. isolates, *S. pseudintermedius* causes urinary tract infections, otitis, wound infection, soft tissue infections, and surgical site infections and is the leading cause of pyoderma in dogs [29,30]. Similar to strains of SA, *S. pseudintermedius* has developed resistance to methicillin, resulting in more complex and costly treatment options for veterinary patients [4,11,31]. It is known that the spread of *S. pseudintermedius* usually involves contact between two hosts. With increased exposure between humans and veterinary patients, the risk of the transmission of pathogens will likely rise, especially with MRSA, antibiotic-resistant SIM, and Cdiff [12,21,32]. Zoonotic pathogens are a huge public health concern, and interspecies transfer of these pathogens between animals and humans could enhance the horizontal exchange of resistance factors between these pathogens [12,21,32]. 

With the close contact between pets and their owners, other animal patients, and veterinary medical personnel in veterinary rehabilitation facilities, it is important to know if surfaces within the facility are contaminated by potential pathogens. Knowledge of problematic surface spots in these clinics should encourage managers of these clinics to proactively clean and disinfect sites known to be contaminated by these pathogens. The aim of this study was to determine the prevalence of contamination by potential pathogens of both humans and animals from environmental surfaces and equipment commonly found in veterinary physical rehabilitation clinics. Overall, we found bacterial contamination by potential pathogens to be commonplace throughout the clinics sampled. Future studies should seek any links between this background contamination and the incidence of HAIs in clinic patients. 

## 2. Materials and Methods

This study involved the collection of bacterial swab samples from environmental surfaces and water samples from underwater treadmill tanks in five different veterinary rehabilitation clinics. Within the clinics, we identified 13 items/locations (Table 1) that were present in each clinic for the collection of swab samples. Sampling involved using double transport swabs (Fisherbrand, with Stuart’s liquid medium, Fisher Scientific, Pittsburg, PA, USA), which were used to sample areas of approximately 100 cm^2^ at each sample site. Both of the two swabs present in these double swabs were carefully brought into contact with the surfaces sampled. For additional information regarding how these swabs were used to inoculate growth medium and how the surface areas of the swabs were estimated, please see the Supplementary Materials File S1. After collection, the swabs were placed on ice and transported to a microbiology lab on the University of Tennessee at Chattanooga (UTC) campus for processing (within four hours of swab collection). This lab is a Biosafety Level 2 certified lab, all personnel working with the samples wore appropriate personal protection equipment (e.g., lab coat, safety glasses, and gloves), and all swab and culture manipulations occurred in a properly functioning biosafety cabinet.

At the lab, these swabs were used to inoculate four different selective and differential media types, one selective enrichment broth, and one non-specific bacterial growth medium. The media used included two from Hardy Diagnostics (Hardy Diagnostics, Santa Maria, CA, USA), as follows: a selective enrichment broth, Hardy Cdiff Banana Broth (Hardy Cat.# K226, selective for the enrichment of *Clostridium difficile* [Cdiff]), and a selective and differential agar, Hardy Diagnostic’s CHROM MRSA agar (Hardy Cat.# G307, selective for methicillin-resistant *S. aureus* [MRSA] and *S. pseudintermedius* [SIM]). We used three selective and differential medium agars from Fisher Scientific (Fisher Scientific, Pittsburgh, PA, USA), including mannitol salt agar (Fisher Cat.# B2127X [BD Mfr.# 221271] for *S. aureus* [SA] and *S. epidermidis* [SE]); eosin methylene blue agar (Fisher Cat.#. B11221 [BD Mfr.# 211221], EMB, for enteric bacteria [ENT]); and pseudomonas isolation agar (Fisher Cat.# DF0927-17-1 [BD Mfr.# 292710] for *Pseudomonas* spp. [PS] and *P. aeruginosa* [PSA]). In addition to the selective and differential media, we also inoculated a non-specific type of bacterial growth media, tryptic soy agar [TSA] (Fisher Cat.# DF0369-17-6 [BD Mfr.# 236950]), which was used to detect *Bacillus* spp. [BAC] and *Micrococcus* spp. [ML]. The inoculation of the Hardy Cdiff banana broth necessitated the use of one of the two double swabs, with that swab being aseptically transferred into the broth tube and clipped off with flame-sterilized scissors to allow the swab to fit inside the tube. The second of the double swabs was then used to inoculate all the selective and differential media and the TSA plates using a line inoculation technique (as described in Keilman et al. [33], please see the Supplementary Materials for more information regarding this line inoculation technique). In short, for the line inoculations, the five agar plates to be inoculated were placed side by side in the biosafety cabinet, and using a gentle, short, 4 cm, straight-line motion, one medium at a time was inoculated. When changing to a new agar type, the swab shafts were rotated approximately 1/5 of a rotation to bring a fresh surface of the swab to the different agar surfaces. Inoculated agar plates and the Hardy banana broth tubes were placed in a 37 °C incubator and incubated for 48 h.

Water samples were collected from the underwater treadmill tanks using a 10 mL pipette with sterile pipette tips, transferring these samples into sterile WhirlPack sample bags (500 mL, Fisher Scientific, Pittsburgh, PA, USA), and after proper sealing, all the sample bags were placed in a cooler on ice for transport back to the microbiology lab at UTC. At the lab, 0.1 mL subsamples were aseptically removed from the sample bags using pipettes with sterile tips and transferred onto the surface of TSA and EMB plates. These aliquots of water samples were spread over the entire surface of the plates using flame-sterilized spreading rods. Plates inoculated with water samples were also placed in a 37 °C incubator and incubated for 48 h. 

The interpretation of bacterial growth on the different media allowed for the observation of different types of bacteria colonies typically found to grow on the specific medium types, leading to our presumptive conclusions. For additional information regarding how different presumptive bacterial identifications were made from incubated media, please see the Supplementary Materials. We were also able to count the number of colonies growing on the plates inoculated with water samples. For the selective and differential media used to indicate staphylococci presence, growth on MSA enabled the detection of both mannitol-positive (for fermentation, turning the medium yellow with white to tan colonies, e.g., SA) staphylococci and mannitol-negative staphylococci (with white colonies leaving the medium red, e.g., SE). Using the CHROM MRSA agar plates, mauve-colored to white with pink streak colonies were known to represent presumptive colonies of MRSA [34]. Using EMB agar plates, pink to dark purple colonies were indicative of lactose fermentation and were used to presumptively identify enteric bacteria [ENT]. For *Pseudomonas* spp. we scored yellowish-green colonies on PSI agar as positive for *P. aeruginosa* [PSA] and white colonies as *Pseudomonas* spp. [PS]. Colonies growing on the TSA plates were used to estimate the overall level of non-specific bacterial colonization of the different sites sampled, specifically allowing for the observation of *Bacillus* spp. [BAC]. or *Micrococcus* spp. [ML]. 

Once bacterial identification on the plates was determined, these data were presented as percent-positive swabs by species and sample site. Data were analyzed using descriptive statistics. The analysis was performed using SPSS 26 (Armonk, NY, USA: IBM Corp).

## 3. Results

**Figure 1:**
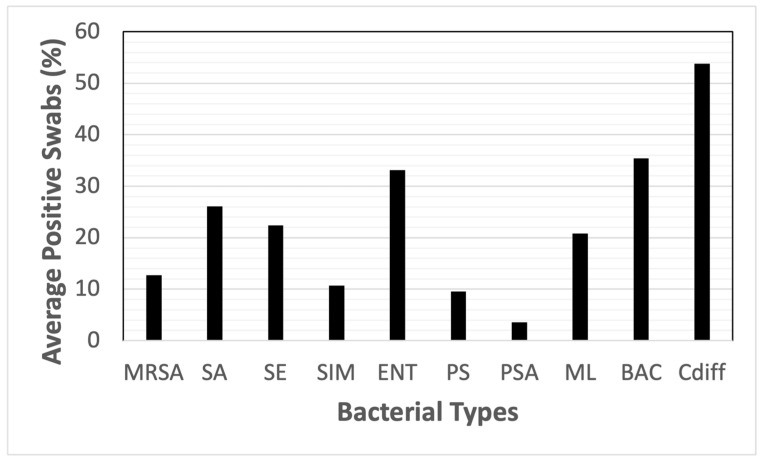
Average of the percent-positive swabs for all sites by bacterial species. Key: MRSA = methicillin-resistant *S. aureus,* SA = *S. aureus*, SE = *S. epidermidis* (mannitol-negative), SIM = *S. pseudintermedius*, ENT = enteric bacteria (lactose-positive, Gram-negative rods), PS = *Pseudomonas* spp., PSA = *P. aeruginosa*, ML = *Micrococcus* spp., BAC = *Bacillus* spp., Cdiff = *Clostridium difficile*.

The most common presumptive contaminating species based on an average of percent-positive swabs for all sites was Cdiff, with BAC and ENT being the next two most common contaminating bacterial types observed. Contamination by Cdiff was found at 58.3% of all sites sampled, while BAC and ENT were found on 35.4% and 33.1% of sites (Figure 1). At the lower end of the contamination range, we found PSA contaminating only 3.6% of sites. 

When looking at clinic contamination by site and species, Cdiff contaminated 94.7% of the floors and 83.3% of the HVAC return air ducts (Table 1). Enteric bacteria and *Bacillus* spp. were the next most encountered contaminants and were found on 100% of swabs from the return air ducts. The floors and return air ducts were consistently contaminated by other species, including Cdiff, SA, MRSA, and SIM. The highest levels of contamination by SA were found on the HVAC return air duct (83.3%) and the scales (83.3%).

**Figure 2:**
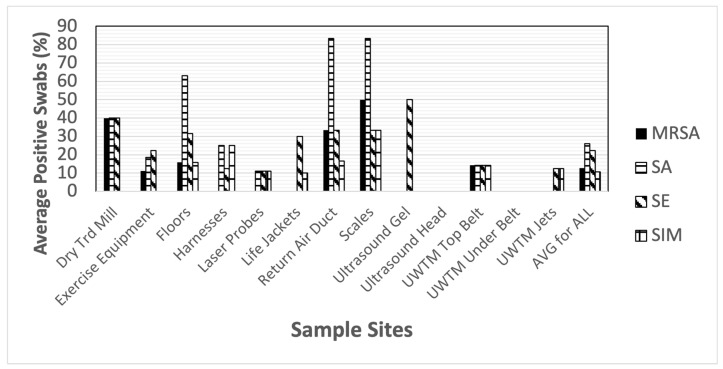
Total positive swabs by site having *Staphylococcus* spp. Presumptive species identification: MRSA = methicillin-resistant *S. aureus*, SA = *S. aureus*, SE = *S. epidermidis*, SIM = *S. pseudintermedius*.

When looking at the presumptive bacterial contamination of different clinic sites, a large range of contamination was observed. Staphylococci were found most prominently on the floors, with SA found on the largest number of swabs (Figure 2). The only sites in which staphylococci were not found were the ultrasound gel bottles and heads and the bottom surface of the belt on the underwater treadmills.

**Figure 3:**
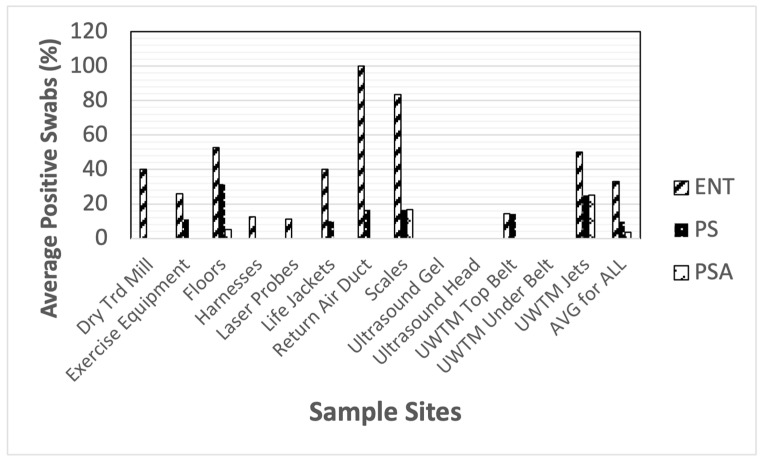
Total positive swabs by site with select Gram-negative bacteria. Presumptive species identification: ENT = enteric bacteria (lactose-positive, e.g., *Escherichia* spp.), PS = *Pseudomonas* spp., PSA = *Pseudomonas aeruginosa*.

Bacterial contamination due to select Gram-negative rods was also found throughout the veterinary clinics. Enteric bacterial contamination (lactose-positive cells, e.g., *Escherichia* spp.) was found on the greatest number of sampled sites of the Gram-negative bacteria studied (Figure 3). Again, the floors were the most contaminated sites in these clinics. *Pseudomonas aeruginosa* was also found in the clinics, but in relatively low numbers. Other species of *Pseudomonas* spp. were generally widespread in the clinics at slightly higher numbers.

**Figure 4:**
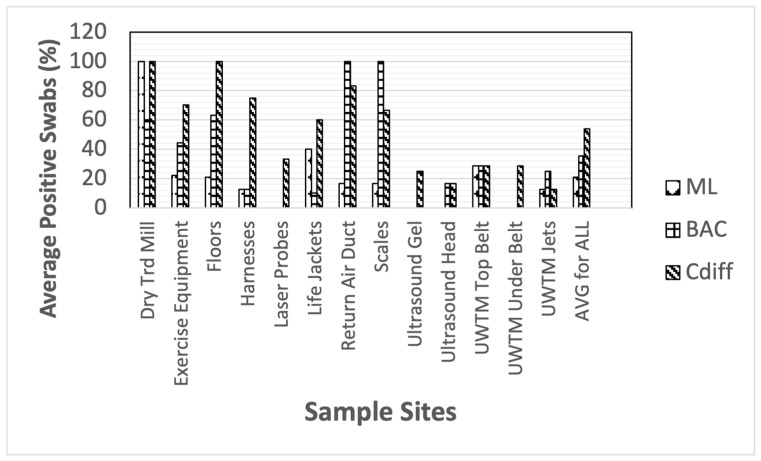
Total positive swabs by site with select Gram-positive bacteria. Presumptive species identification: ML = *Micrococcus* spp., BAC = *Bacillus* spp., Cdiff = *Clostridium difficile*.

Bacterial contamination by other select Gram-positive species was also found throughout the clinics. Most notably, Cdiff was found in a very high number of sites throughout the clinics (Figure 4). Another spore-forming genera of Gram-positive bacteria, *Bacillus* sp., was also found contaminating many of the same sites as Cdiff. *Micrococcus* spp., a Gram-positive coccus often associated with human skin, was also found on many sites throughout the clinic. 

**Figure 5:**
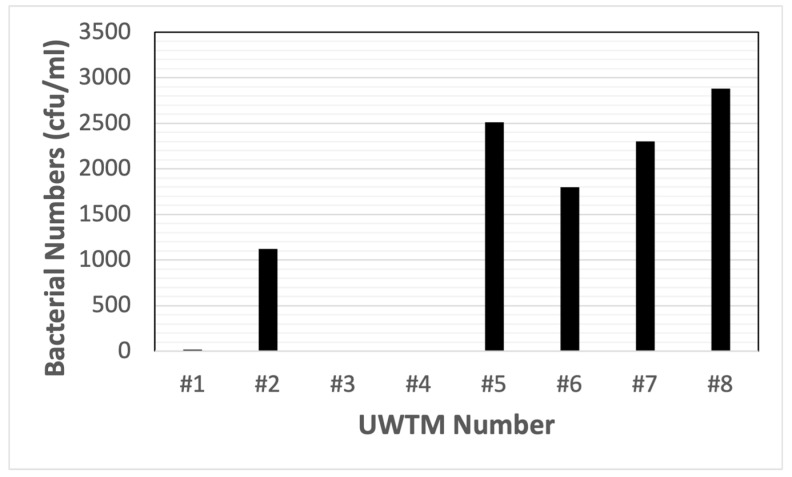
Water sample contamination for water from underwater treadmill (UWTM) tanks at five veterinary clinic sites. Individual UWTMs are indicated by number (#1 through #4 were at one clinic, while #5 to #8 were each at different clinics). Total colony counts (per mL) for TSA and EMB media streaked with 0.1 mL samples from the treadmill tanks.

When water from underwater treadmill tanks was streaked onto TSA and EMB agar plates, colony counts as high as 2800 cfu/mL were detected (Figure 5). The largest number of bacterial colonies observed was for enteric bacteria.

## 4. Discussion

This study assessed the patterns of bacterial contamination on medical equipment and environmental sites in veterinary physical rehabilitation clinics. Bacterial species that were isolated and grown included both Gram-negative and Gram-positive bacteria. Of the Gram-positive cocci, several *Staphylococcus* spp. were observed, including SA, MRSA, SE, and SIM. Of the Gram-negative bacteria detected, enteric bacteria (e.g., *Escherichia* spp.) and several species of *Pseudomonas* were represented. In general, the veterinary clinics surveyed were largely contaminated by Gram-positive bacteria, with presumed staphylococcal contamination being the most frequent throughout the sampled sites. Notably, *S. pseudintermedius* can be problematic for both dogs and humans, possibly causing disease in both species [35]. Two of the Gram-positive rods detected, Cdiff and BAC, are spore-forming species. This is important since bacterial spores offer the species a higher degree of resistance to many abiotic factors that may be employed to control bacterial contamination. In addition, many *Bacillus* spp. are associated with soil, and since dogs are known to dig in soil quite often, veterinary patients may carry these bacteria into the clinics on their skin or feet. The other spore-forming bacteria, Cdiff, is a notable human pathogen and could pose a threat to care-giving humans in these clinics [9,10,36]. 

Within the clinics, several “hot spot areas” having high levels of bacterial contamination, such as HVAC return air ducts, floors, and scales, are common to veterinary patients and represent sites with which human caregivers also interact. As the results demonstrate, these areas had 50–94.7% positive swabs for multiple potential pathogenic bacteria. Of the bacteria cultured from HVAC return air ducts and from the floors, spore-forming bacteria (e.g., Cdiff and BAC) were dominant. When bacterial species that may be from soil are found on both floors and HVAC return air ducts, this suggests that levels of dust in the facilities might be high. When dust is suspended in the air, it can help transmit airborne pathogens. Airborne pathogens in human hospitals have been found to contribute to infections in both patients and their caregivers [37]. Targeted cleaning and disinfection of veterinary clinics to reduce dust may be a good strategy to reduce the potential for contamination of animals and their caregivers. 

Water in the underwater treadmill tanks was contaminated by both enteric and non-specific bacteria. If the enteric bacteria observed were of fecal origin, dogs using the treadmills may be contaminated by a wide range of fecally transmitted dog pathogens [38]. One measure that can be used to reduce waterborne contamination in underwater treadmills is to use shock treatments with chlorine-, bromine-, or hydrogen peroxide-based chemical treatments. Periodic emptying of the tanks and refilling with treated water (saltwater system or low levels of bromine or chlorine) may help reduce the potential for contamination with enteric pathogens. 

A recent study performed by Lord et al. found increased numbers of antimicrobial-resistant bacteria in hospitals, particularly *Staphylococcus* spp. [4]. In some cases, resistance against last-tier antimicrobial therapies (e.g., fluoroquinolones and phenicols) was observed in strains of *Staphylococcus* spp. [4,39,40]. Although methicillin-resistant *S. pseudintermedius* was not a focus of this study, it is highly probable that a horizontal exchange of resistant factors between strains of MRSA and this dog pathogen could occur [41]. Antimicrobial and multidrug-resistant pathogens are a huge public health concern to both veterinary patients and their caregivers and need to be addressed in all clinics.

This study was based on the use of viable bacterial culture techniques to monitor contamination in the veterinary clinics sampled. Many contemporary studies of the presence of bacteria (and other pathogens) in human health care facilities use some form of a molecular approach to extract DNA from samples and determine the diversity of the microorganisms present [42]. Culture techniques provide useful data with regard to the presence of viable cells on surfaces from the sampled sites. Using molecular data to describe the bacterial diversity of a site provides little or no evidence of whether the cells are viable. There is no evidence that DNA from *Staphylococcus* spp. alone causes HAIs; however, viable staphylococci could cause HAIs. Thus, the data presented here represent living contaminants present on clinical surfaces that could easily cause infections in open wounds or other sites on animal patients. Measures to address contamination in these clinics should be a priority. 

Potential limiting factors related to this study fall into two areas. The first is any problems associated with the line inoculation procedure. The second is the possibility of colonial bacterial growth on the different media for species that should have been selected against, possibly confusing the identification of the species the medium is selective for. In this study, since we were only inoculating five agar-based media, we had to rotate the swab somewhere between 14% and 20% of a full rotation (see Supplementary Materials for additional details) to ensure that a fresh surface of the swab came into contact with the next agar surface being inoculated. Rotating the swabs more (or less) than that may not result in the use of a fresh swab surface to inoculate all of the media being inoculated with that swab. Another possible issue with the use of our line inoculation procedure would be the absence of colonies from a bacterial species present in very low numbers on the swab. If the numbers of this species are so low that there are not at least five cells uniformly distributed around the swab, then we may detect a species on one medium but not another in the series of media inoculated. Another concern with the use of this technique is the potential for the growth of species that may confound the identification usually made on the selective media. For example, on MSA, we know that several non-staphylococci species have been found to survive. According to the Beckton, Dickinson Co. [43] in a quality control report on their MSA medium, *Proteus mirabilis* (Gram-negative) shows “partial” growth on this medium. Another non-staphylococci species that will grow on MSA is *Bacillus subtilis*, which is tolerant of high salt concentrations. Because there are bacterial species that can grow on selective and differential media that should inhibit their growth, possible inhibition of the growth of the desired species on that medium may occur. A recent study has found that *B. subtilis* can produce a bacteriocin capable of the inhibition of *S. aureus* and some enteric bacteria [44]. If there are other such amensalistic interactions between different bacterial species on our selective and differential media, then results generated using the line inoculation procedure might miss the inhibited species. Overall, we feel that these limitations are not critical enough to change our presumptive identifications of the bacteria we identify, which should give clinic managers data to work with in their cleaning and disinfection procedures.

Areas of the veterinary clinics studied here that need to have focused cleaning and disinfection include the HVAC return air vents, scales, exercise equipment, and floors. Although ultrasound coupling gel can be obtained in sterile packets, the clinics studied use gel in reusable bottles. A previous study of human physical therapy clinic ultrasound devices found that the tips of coupling gel bottles were often contaminated with MRSA [45]. Scales are frequently used by all patients entering the clinic and increase the risk of acquiring pathogens that may result in an infection. All these factors increase the risk to veterinary patients and their human caregivers and warrant further investigation and care with disinfection.

## 5. Conclusions

These findings suggest that veterinary clinical environmental surfaces and water are generally contaminated by both Gram-positive and Gram-negative bacterial species. Some of the presumptive contaminating species of bacteria are known pathogens of both dogs and humans (e.g., MRSA, SA, PSA) or specifically pathogenic to humans (e.g., Cdiff). This contamination has the potential to contribute to HAIs that may occur in veterinary patients or their human caregivers. Further research is warranted to investigate the extent of bacterial contamination in veterinary clinics and any potential links to HAIs occurring in animals being treated in those clinics. 

## Figures and Tables

**Table 1:** Percentage of positive swabs having viable bacteria by site and bacterial type or species. Legend: UWTM = underwater treadmill; exercise equipment = peanuts, balance boards, physiorolls, donuts, etc.; MRSA = methicillin-resistant *S. aureus,* SA = *S. aureus*, SE = S. epidermidis (mannitol-negative), SIM = *S. pseudintermedius*, ENT = enteric bacteria (lactose-positive, Gram-negative rods), PS = *Pseudomonas* spp., PSA = *P. aeruginosa*, ML = *Micrococcus* spp., BAC = *Bacillus* spp., Cdiff = *Clostridium difficile*.

	MRSA	SA	SE	SIM	ENT	PS	PSA	ML	BAC	Cdiff
Dry Treadmill (belt, *n* = 5)	40%	40%	40%	0%	40%	0%	0%	100%	60%	80%
Exercise Equipment (*n* = 27)	11.1%	18.5%	22.2%	0%	25.9%	11.1%	0%	22.2%	44.4%	74.1%
Floors (*n* = 19)	15.8%	52.6%	31.6%	15.8%	52.6%	31.6%	5.3%	21.1%	63.2%	94.7%
Harnesses (*n* = 8)	0%	25%	12.5%	25%	12.5%	0%	0%	12.5%	12.5%	75%
Laser Probes (tip of probe, *n* = 9)	0%	11.1%	11.1%	11.1%	11.1%	0%	0%	0%	0%	33.3%
Life Jackets (*n* = 10)	0%	0%	30%	10%	40%	10%	0%	40%	10%	60%
Return Air Ducts (*n* = 6)	15.8%	83.3%	33.3%	16.7%	100%	16.7%	0%	16.7%	100%	16.7%
Scales (*n* = 6)	50%	83.3%	33.3%	33.3%	83.3%	16.7%	16.7%	16.7%	100%	66.7%
Ultrasound Gel (bottle tip, *n* = 4)	0%	0%	50%	0%	0%	0%	0%	0%	0%	25%
Ultrasound Heads (*n* = 6)	0%	0%	0%	0%	0%	0%	0%	0%	16.7%	16.7%
UWTM Top Belt (*n* = 7)	14.3%	14.3%	14.3%	14.3%	14.3%	14.3%	0%	28.6%	28.6%	28.6%
UWTM Bottom Surface of Belt (*n* = 7)	0%	0%	0%	0%	0%	0%	0%	0%	0%	28.6%
UWTM Jets (inside surface) (*n* = 8)	0%	0%	12.5%	12.5%	50%	25%	25%	12.5%	25%	12.5%

## Data Availability

We intend to make data generated in this study available via a cloud-based system provided by the University of Tennessee.

## Supplementary Materials

The following supporting information can be downloaded at https://www.mdpi.com/article/10.3390/ani14131896/s1, File S1: Methods used for Presumptive Bacterial Identification Using Swabs Collected from Clinic Surfaces.
