# Supplementary material for: Bacterial Contamination of Environmental Surfaces of Veterinary Rehabilitation Clinics
Source: Animals (Basel). 2024 Jun 27;14(13):1896. doi: 10.3390/ani14131896 (PMC11240356; doi:10.3390/ani14131896)
Supplement: Supplementary file 1 [file animals-14-01896-s001.zip › animals-3015406-supplementary.pdf]

Supplementary Materials File S1: Methods used for Presumptive Bacterial Identification Using Swabs Collected from Clinic Surfaces

Estimations of Swab Areas used for Line Inoculations

We have developed a quick "line inoculation" technique that allows the use one swab (of the two present in the Fisherbrand "double" transport swabs, see Materials and Methods section for details) to inoculate the five different agar-based media types we use here. Note that the sterile swabs used are "transport" swabs which are pre-wetted with Stuart's liquid medium, which helps stabilize natural communities of bacteria on the swab surface. To verify that the use of one of our swab samples to inoculate five different agar surfaces gave equal distribution of cells on the different media surfaces we first had to estimate the percentage of the entire surface area of the swabs that came in contact with the agar via the 4 cm line inoculations. In the lab we used a fine-pointed marker pen to place a small mark on one side of a test swab's shaft (a sterile swab) near the swab end. Holding that marked side of the swab upwards, we then touched the center of a clean glass microscope slide and rolled the swab across the surface of the slide one full revolution until the mark on the shaft returned to the original, upwards starting position. After completing this rolling motion, we quickly touched the tip of the swab to an unused end of the glass slide. The intention was to bring the entire surface area of the swab that could come in contact with either the sample surface or the agar surface into contact with the glass slide. Immediately after touching the glass slide with this wet swab, we took a glass maker pen and (on the bottom of the glass slide) made an outline of the areas of the glass slide that were wet due to coming into contact with the swab. The total surface area of the swabs was then calculated from the areas marked by the pen on the backside of the slide in mm^2. To estimate the area of a swab that was brought into contact with the agar surface, a clean glass slide was used where a sterile swab was placed on the center of the slide and gently pressed into the glass slide using the same amount of force used to press a sample swab onto an agar surface for the 4 cm inoculation streak. This glass slide was also marked with a pen and the wet area determined in mm^2. This process was repeated with three test swabs for the surface area, and then three additional swabs for the surface area of swabs used for inoculation. The estimated total surface area of these swabs was 355 mm^2, while the area of one side of the swab that made contact with the agar surface was 50 mm^2. Thus, the careful application of line inoculations on one agar surface should account for only approximately 14% of the swab's surface area. As long as the swab sample collection provides brings all parts of the surface of the swab in contact with the environmental surface, then by rotating the swab about 15%-20% in the same direction between each agar inoculation a fresh swab surface should provide a representative sample for inoculation of the five different agar types used here.

The purpose of this study was to simply provide data on the presence or absence of presumed bacterial types from the swabs collected. We do not make any attempt at estimating relative numbers of cells on the surfaces we sampled. It is possible that if a species of bacteria were present on the swabs (e.g., on the environmental surfaces) in very low numbers, then we might not get uniform distribution of those cells on the five different agar-based media we used. If we were attempting to quantify the numbers of cells present on the surfaces then this issue would require additional verification with lab cultures in very low numbers.

Interpretations of Bacterial Growth After Incubation

To interpret colonial growth on the different types of media we inoculated we used both personal experience gained by having analyzed large numbers of sub-cultured colonies from similar studies of the environment of human clinics over the past five years, as well as recommendations from the manufacturers of the media used for the interpretation of colony growth on their media. Because we are not a diagnostic microbiology lab, our interpretations are presumptive only. Further sampling and analyses would be necessary to provide bacterial identifications of a diagnostic nature. Because we provide a rapid response to the collection of large numbers of swabs from clinic surfaces, the utility of making presumptive identifications for groups of pathogens is important. In addition to using a line inoculation technique (see above) that allowed us to inoculate five different agar-based medium types plus one broth medium (the Hardy Cdiff Banana broth) we were able to obtain growth on all of the media after 48 hours of incubation. After incubation we interpreted the growth on each of the different medium types. Our research personnel have gained previous experience presumptively identifying the bacteria we identify by using prior data from clinical studies, along with manufacturer's recommendations. In these previous studies we subcultured approximately 10% of the colonies (over 500 colonies) that grew on agar-based media we isolated from other clinics over about a five-year period.. For all of the sub-cultured colonies obtained we subjected them to further analyses to verify our presumptive identities based on standard microbiological criteria that included colonial morphologies, staining reactions, reactions on selective and differential media, and certain differential tests.

The cultural characteristics we observed for our sub-cultured colonies included: colony morphology (including colors), Gram stain reaction, Spore stain reaction (for Gram positive rods), and some additional specific testing. For example, for a select group of about 50 sub-cultured colonies that showed the characteristic mauve-colored to white with pink streaked colonies on Hardy CHROM MRSA agar, which data from Flayhart et al. [34] suggest are specifically methicillin resistant *S. aureus* (MRSA), a colleague in a UTC lab ran polymerase chain reaction studies (data not shown) searching for the *mec*-A gene in these sub-cultures. The *mec*-A gene is found in many staphylococci that are resistant to methicillin and related antibiotics. Results of the colleague's work indicated that our sub-cultured colonies from Hardy CHROM MRSA medium that had mauve-colored to white with pink streaked colonies all possessed the *mec*-A gene. Along with other characteristics we obtained in our lab (e.g., Gram positive cocci, coagulase positive, DNAse positive) we can make a presumptive identification of MRSA from the results on the Hardy CHROM MRSA medium.

For other presumptive cultural identifications on different types of growth media utilized in this study we used the following characteristics for our presumptive identifications - For *Clostridium difficile* [Cdiff] , we use the Hardy Cdiff Banana enrichment recovery broth to indicate the presence of Cdiff. This medium will turn from red to yellow when Cdiff is present, which is how we scored positive Cdiff samples. For Staphylococci, we also use the mannitol salt agar (MSA) medium, where abundant colonial growth is found for staphylococci, which are not sensitive to the 7.5% concentration of NaCl in the medium. Staphylococci are further differentiated on MSA via their abilities to ferment the sugar mannitol that is added at 10% to the medium.

Acid generated when the mannitol is fermented changes the medium color from red to yellow. *Staphylococcus aureus* [SA] will grow with white to tan colored colonies from 2 mm to 5 mm diameter on MSA and will turn the medium yellow due to fermentation of mannitol. *Staphylococcus epidermidis* [SE] will produce white colonies on MSA, also 2 mm to 5 mm diameter, leaving the medium red due to the culture's inability to ferment mannitol. Other characteristics we have used to verify staphylococci from the MSA sub-cultured colonies include Gram stain (staphylococci are all Gram-positive cocci), as well as other tests including coagulase tests (SA is positive, SE is negative), and DNAse tests (SA is positive, SE is negative). For enteric bacteria [ENT] we use results from the eosin methylene blue (EMB, Levine type) agar medium, where pink to purple, mucoid colonies represent lactose positive fermentation characteristic of coliform type enteric bacteria. We have verified these results on sub-cultures by performing Gram stains (ENT are all Gram-negative rods) and running lactose fermentation tests, with ENT being positive fermenters. Growth on EMB agar included the lactose fermenters noted, but also will allow notable lactose non-fermenters such as *Salmponella* spp. and *Shigella* spp. to grow with colorless colonies. Enterococci spp. sometimes exhibit limited growth on EMB. Some *Bacillus* spp. may also grow in a limited fashion on EMB, showing opaque, granular colonies. For *Pseudomonas* spp., we use *Pseudomonas* isolation agar (PIA) where *Pseudomonas* spp. [PS] will grow with white colonies, while Pseudomonas aeruginosa [PSA] grows with yellow to green colonies. Other Gram-negative cells (e.g., *Escherichia* spp.) tend to be completely inhibited on PIA agar. *Pseudomonas* spp. and PSA are both Gram-negative rods, and lactose negative fermenters.

For *Bacillus* spp. [BAC] and *Micrococcus* spp. [ML] we do not use selective and differential media for our presumptive identifications. We use well established colonial morphologies for these bacteria when grown on tryptic soy agar (TSA). The colony characteristics we seek to presumptively identify BAC include large opaque, irregular, dry/friable, granular colonies that are >8 mm diameter, cream to tan color. The Gram-stain reaction of BAC yield Gram-positive rods. We have also run spore stains on BAC colony types and find them to be spore-positive rods. For ML colony characteristics we include medium sized opaque, circular, butyrous colonies between 2 and 5 mm diameter, yellow to tan color. The Gram-stain reaction of ML is Gram-positive cocci that appear to be larger than staphylococci.

All of the culture identifications we make here are presumptive in nature. If a veterinarian needs more certainty in the species we identify, the use of specific diagnostic laboratories capable of determining bacterial species with certainty will be required.
